# The stabilization of yes‐associated protein by TGFβ‐activated kinase 1 regulates the self‐renewal and oncogenesis of gastric cancer stem cells

**DOI:** 10.1111/jcmm.16660

**Published:** 2021-06-01

**Authors:** Gang Wang, Qikai Sun, Hai Zhu, Yihui Bi, Haixing Zhu, Aman Xu

**Affiliations:** ^1^ Department of General Surgery The Fourth Affiliated Hospital of Anhui Medical University Hefei China; ^2^ Department of Hepatobiliary Surgery The Affiliated Drum Tower Hospital of Nanjing University Medical School Nanjing China; ^3^ Department of General Surgery The First Affiliated Hospital of Anhui Medical University Hefei China; ^4^ School of Pharmacy Anhui Institute of Innovative Drugs Anhui Medical University Hefei China; ^5^ Department of Gastrointestinal Surgery Anhui Provincial Cancer Hospital Hefei China

**Keywords:** cancer stem cells, gastric cancer, TGFβ‐activated kinase 1, YAP

## Abstract

Gastric cancer (GC) is the most frequent digestive system malignant tumour and the second most common cause of cancer death globally. Cancer stem cell (CSC) is a small percentage of cancer cells in solid tumours that have differentiation, self‐renewal and tumorigenic capabilities. They have an active participation in the initiation, development, metastasis, recurrence and resistance of tumours to chemotherapy and radiotherapy. Gastric cancer stem cells (GCSCs) have been shown to be correlated with GC initiation and metastasis. In this study, we found that TAK1 expression level in GC tissues was significantly increased compared to the adjacent non‐cancerous tissues by RT‐qPCR, Western blot and immunohistochemistry. TAK1 has been identified as a critical molecule that promoted a variety of malignant GC phenotypes both in vivo and in vitro and promoted the self‐renewal of GCSCs. Mechanistically, TAK1 was up‐regulated by IL‐6 and prevented the degradation of yes‐associated protein (YAP) in the cytoplasm by binding to YAP. Thus, TAK1 promoted the SOX2 and SOX9 transcription and the self‐renewal and oncogenesis of GCSCs. Our findings provide insights into the mechanism of self‐renewal and tumorigenesis of TAK1 in GCSCs and have broad implications for clinical therapies.

## 1 INTRODUCTION

Gastric cancer (GC) is the most frequent malignant neoplasm of the human digestive system. In 2018, it had the sixth highest incidence rate among all types of cancer worldwide and the second highest mortality rate.^1^ Currently, surgical resection is the only possible way to cure GC. However, most patients are already in late stage at the time of first diagnosed or during treatment. Besides, unsatisfactory surgical results can cause post‐operative recurrence and metastasis.^2^ In addition, GC patients are prone to show chemotherapy resistance after surgery.^2^ Although cancer stem cells (CSCs) represent only a short tumour cell fraction, they are closely associated with tumour occurrence, recurrence, metastasis and chemotherapy resistance.^3^ CSC occurrence have been confirmed in several tumour types, including gastric cancer stem cells (GCSCs).^4^, ^5^, ^6^, ^7^ GCSCs can be characterized by several biomarkers, such as CD44,^7^ Lgr5,^8^ CD133^9^ and CD90.^10^ Furthermore, GCSCs have been considered a relevant subset of targets for an efficient GC treatment. However, the GCSCs mechanism of action in GC has not yet been clarified.

TGF‐β‐activated kinase 1 (TAK1) is a mitogen‐activated protein kinase kinase that acts on nuclear factor κB (NF‐κB) and activator protein‐1 (AP‐1) activation. TAK1 plays essential roles in several biological responses, such as cell survival, development, metabolism, carcinogenesis, immune responses and chemoresistance.^11^, ^12^ Several studies have shown that TAK1 exerts a key role in tumour initiation, progression and metastasis and can behave as a tumour promoter^13^, ^14^, ^15^, ^16^ or suppressor.^17^, ^18^ Current evidence indicates that TAK1 performs a fundamental function in the stem cell regulation.^19^, ^20^ Furthermore, TAK1 has been described to be highly expressed in GC tissues and that may be associated to the GC occurrence and development.^21^ Nevertheless, the detailed molecular mechanism by which TAK1 acts in GC remains elusive.

Recent advances highlight the Hippo pathway role in the tissue regeneration, organ development, stem cell self‐renewal and tumorigenesis.^22^, ^23^ The main Hippo kinase cascade function is to supress the oncogenic transcriptional complex formed by the yes‐associated protein (YAP), the transcriptional co‐activator with PDZ‐binding motif (TAZ) and TEA domain family members (TEAD). YAP/TAZ act as transcriptional activators that translocate between the nucleus and the cytoplasm and lead to expression of target genes by interaction with transcription factors of the TEAD family. Different studies have indicated that YAP and TAZ perform crucial roles in maintaining CSCs and cancer progression.^24^, ^25^, ^26^, ^27^

In this study, TAK1 has been identified as a tumour promoter positively correlated with poor prognosis and recurrence in GC. We demonstrated that TAK1 is up‐regulated by IL‐6 in the cell microenvironment and binds to YAP, thereby promoting self‐renewal by regulating SOX2 and SOX9 in GCSCs.

## 2 MATERIALS AND METHODS

### 2.1 Clinical tissue samples

Gastric cancer (GC) and relative adjacent non‐cancerous samples and detailed clinical and pathological data (including gender, age, drink, tumour size, location and differentiation, and T, N and TNM stages) were acquired from the clinical records of 200 patients diagnosed at The Fourth Affiliated Hospital of Anhui Medical University (Hefei, China) between January 2014 and December 2019. The samples were selected for this study on the basis of TNM staging system from the Union for International Cancer Control (8th edition). Patients who received chemotherapy or radiotherapy before surgery were excluded. The samples were fixed in a 4% formalin solution at 37°C for 2 hours and paraffin‐embedded for pathological analysis and diagnosis confirmation. Patient clinical follow‐up data were acquired from the GC database of The Fourth Affiliated Hospital of Anhui Medical University. All procedures in this study have been endorsed by the Ethics Committee of The Fourth Affiliated Hospital of Anhui Medical University (certification no. 20150232) and were carried out in accordance with the rules put forward in the Declaration of Helsinki. All patients signed an informed consent form.

### 2.2 Cell culture and cell lines construction

Five human GC cell lines (MKN45, MGC803, MKN28, HGC27 and AGS) and a normal gastric mucosal cell line (GES‐1) were obtained from the Cell Bank of the Chinese Academy of Sciences (Shanghai, China). All cells were cultured in RPMI‐1640 or DMEM (BI, Haemek, Israel) containing 10% foetal bovine serum (BI, Haemek, Israel) and 100 U/mL penicillin‐streptomycin (Gibco; Thermo Fisher Scientific Inc) in an atmosphere containing 5% CO_2_ at 37°C. They were authenticated by STR profiling and tested to be pathogen and mycoplasma negative before the experiments (BioWing Biotechnology). Sh1‐TAK1, sh2‐TAK1, shYAP, sh‐NC, 3 × Flag‐Vector, 3 × Flag‐TAK1 and 3 × Flag‐YAP were synthesized by GenePharma Co. Ltd. The corresponding nucleotide sequences are listed in Table S1. Knockdown and overexpression stable cell lines in the above‐mentioned were established through lentiviral transduction. The established stable cell lines were attested by quantitative reverse transcription‐PCR (RT‐qPCR) and Western blot analyses and used for subsequent experiments.

### 2.3 RNA Extraction and Quantitative real‐time PCR (qPCR)

TRIzol reagent was used to remove total RNA from cells according to the manufacturer's instructions (Invitrogen; Thermo Fisher Scientific Inc). The first‐strand cDNA was synthesized using a cDNA Reverse Transcription Kit (Applied Biosystems; Thermo Fisher Scientific Inc). QPCR was conducted employing the SYBR Green PCR Master Mix (Applied Biosystems; Thermo Fisher Scientific Inc.) to detect the transcription levels. Expression levels were calculated in relation to the GAPDH control. The primer pairs used in the SYBR Green reactions are listed in Table S2. The expression relative levels were determined using the 2^−ΔΔ^ *^C^* ^q^ method.

### 2.4 Western blot (WB) analysis

For the protein expression level detection, WB was conducted. In brief, the cells were lysed using RIPA cell lysis buffer (cat. no. P0013B; Beyotime) containing 1 mmol/L phenylmethylsulphonyl fluoride at 4°C for 30 minutes, with vortexing each 10 minutes, followed by centrifugation at 13 800 *g* for 10 minutes at 4°C. The protein concentration was quantified using a BCA kit (cat. no. P0009; Beyotime). Twenty micrograms of denatured protein for every sample were submitted to a 10% SDS‐PAGE gel electrophoresis and transferred to a PVDF membrane. Non‐fat milk (5%) in TBS buffer containing 0.05% Tween 20 (TBST) was used for membrane blocking for 2 hours at room temperature. Subsequently, the membranes were incubated with the following primary antibodies:

TAK1 (cat. no. ab109526, 1:1000, Abcam); YAP (cat. no. 4912, 1:1000, CST); p‐YAP (cat. no. 4911, 1:1000, CST); LATS1 (cat. no. 3477, 1:1000, CST); LATS2 (cat. no. 5888, 1:2000, CST); 3 × Flag (cat. no. 20543‐1‐AP, 1:500, Proteintech); SOX2 (cat. no. 2748, 1:1000, CST); SOX9 (cat. no. 82630, 1:800, CST); β‐actin (cat. no. 60008‐1‐Ig, 1:2000, Proteintech); GAPDH (cat. no. 10494‐1‐AP, 1:5000, Proteintech). After TBST washing, the membranes were incubated with the secondary antibody (anti‑mouse or anti‑rabbit) for 1 hour at room temperature. After three washes, the membranes were analysed using an enhanced chemiluminescence system (cat. no. P0018AM; Beyotime).

### 2.5 Immunohistochemistry staining

Immunohistochemistry (IHC) staining was conducted using a standard methodology based on streptavidin‐biotin‐peroxidase complex according to the manufacturer's instructions (SA2010; Boster Biological Technology). A moist chamber was used to incubate the tissue sections overnight at 4°C in the presence of anti‐TAK1 antibody (cat. no. ab109526, 1:200, Abcam). TAK1 expression levels were determined by establishing the percentage of positive tumour cell percentage and the positive staining intensity. The intensity of the staining was classified as follows: negative, 0; weak, 1; moderate, 2; and strong, 3. In addition, staining was evaluated according to the stained tumour cell percentage in the field of view as follows: negative, 0; 0%‐25%, 1; 26%‐50%, 2; 51%‐75%, 3; and 76%‐100%, 4. The product of the intensity staining score and stained cell percentage represented the overall IHC score (0‐12). For statistical analysis, scores of 0 to 7 were considered low expression and scores of 8 to 12 were considered high expression. Two independent pathologists accompanied and assessed the staining procedure and results.

### 2.6 Cell proliferation, migration and invasion assays

Cell proliferation capacity was assessed by the clone formation assay. Approximately 2.0 × 10^2^ cells per well were grown in 6‐well plates containing DMEM with 10% FBS at 37°C. The cells were treated with a 10% formaldehyde solution and stained with 0.1% crystal violet (Sigma) after two weeks of culture. The calculation of the colonies formed was performed using a camera (Olympus). The cell migration ability was assessed by scratch wound assay. Approximately 4.0 × 10^5^ cells were seeded in 6‑well plates and cultured overnight to reach >80% confluence in an atmosphere containing 5% CO_2_ at 37°C. Subsequently, a 200‐μL pipette tip was used to make a longitudinal scratch in the centre of the bottom of the sample well. The detached cells were washed away with PBS and followed by the addition of serum‑free medium. The images were captured 0 and 36 h after the wound to examine healing using a light microscope (IX81, Olympus) at 4× magnification. The cell invasion ability was assessed by transwell assay (cat. no. 354480, BD Biosciences). Briefly, about 1.0 × 10^5^ cells in serum‑free medium were added to the upper culture chambers that had been pre‐coated with Matrigel at 37°C. The culture chambers bottom were filled with DMEM complemented with 10% FBS by volume. After 36 h incubation in 5% CO_2_ at 37°C, the invading cell staining was conducted by incubation with 0.5% crystal violet for 10 minutes at room temperature and evaluated using a light microscope (IX81, Olympus) at 10× magnification.

### 2.7 Spheroid‐formation assay

Adherent tumour cells were harvested using trypsinization, and single cells were resuspended with DMEM/F12 (DMEM/F12, BI, Haemek, Israel) medium containing 4 μg/mL heparin (Heparin sodium salt, Sigma‐Aldrich), B27 (cat. no. C11330500BT, B‐27 Supplement (50×), serum free, Gibco), 20 ng/mL human recombinant epidermal growth factor (cat. no. AF‐100‐15, Animal‐Free Recombinant Human EGF, PeproTech) and 20 ng/mL human recombinant basic fibroblast growth factor (cat. no. 100‐18B, Recombinant Human FGF‐basic, PeproTech) after centrifugation. Single cell suspensions were grown on 6‐well ultralow attachment plates (ultralow attachment plates, Corning) at a concentration of 3000 cells/mL at 37°C for 10‐14 days. Then, the spheres were photographed using a microscope and counted with ImageJ cell counter.

### 2.8 Flow cytometry and cell sorting

Surface markers, including CD44 (cat. no. 338804, BioLegend) and CD133 (cat. no. 372806, BioLegend), were assessed by flow cytometry. The cells were treated with trypsin and washed twice with PBS. Then, they were suspended with 100 μL PBS in order to reach a concentration of 1 × 10^7^/mL. Subsequently, suspensions were administered with the antibodies at the standard concentration. After incubation in the dark for 30 minutes at 37°C, the suspensions were washed with PBS and resuspended by 200 μL of the same buffer. The samples were determined and sorted by FAC‐SCantoTM II flow cytometer (BD Biosciences), and the data were examined using FlowJo software (Tree Star).

### 2.9 Binding mode of TAK1 and YAP proteins

The crystal structure of protein TAK1 was obtained from Protein Data Bank (PDB ID: 2EVA). As the YAP crystal structure has not yet been solved, the three‐dimensional structure of the YAP Pro101‐Gln200 region was predicted ab initio using the online QUARK algorithm.^28^, ^29^ The protein‐protein binding mode of TAK1 and YAP was predicted using the HDOCK server.^30^ The residues involved in the interactions between TAK1 and YAP were showed by LigPlot^31^ and PyMOL software (The PyMOL Molecular Graphics System, version 2.0 Schrödinger, LLC.).

### 2.10 Co‑culture of GC cell lines and cancer‐associated fibroblasts

Cancer‐associated fibroblasts (CAFs) were obtained from human GC tissue and normal fibroblasts (NFs) from the non‐cancerous region at least 5 cm from the outer margin of the tumour in the same patient. MKN45 and MGC803 GC cell lines were cultured at 6‐well plates bottom at 10^5^ cells per well. Subsequently, the CAFs were cultured on the upper insert membrane of the transwell chamber (0.4 μm pore size) (Corning Inc.). The cells were incubated for 48 hours at 37°C to evaluate the TAK1 level.

### 2.11 *In* *vivo* experiments

Male six‐week‐old BALB/c nude mice weighing around 18.30 g were acquired from the Experimental Animal Center of Anhui Medical University (Hefei). The nude mice were kept at 20‑26°C, 40‑70% humidity, a 12/12 light/dark cycle, in a pathogen‑free environment and with free access to water and food. For the subcutaneous transplantation model, the mice were implanted with sh‑TAK1‑luciferase or sh‑NC‑luciferase GC cells (2.0 × 10^6^) in the right groin. The mice were killed 4 weeks after implantation, and the tumour volume was calculated. For the tail vein xenograft model, the mice in every group were administered with sh‑TAK1‑luciferase or sh‑NC‑luciferase GC cells (2.0 × 10^6^ suspended in 200 μL PBS) by the tail vein and killed 5 weeks later. Lung nodules and progression were monitored and quantified by bioluminescence. For tumour drug resistance evaluation, each group received an intraperitoneal injection of 5‐FU (cat. no. A4071, 40 mg/kg bodyweight, ApexBio) or cisplatin (cat. no. A8321, 4 mg/kg bodyweight, ApexBio) twice a week for 3 weeks. All procedures with animals have been endorsed by the Ethics Committee of The Fourth Affiliated Hospital of Anhui Medical University (certification no. LLSC20150234).

### 2.12 Haematoxylin and eosin (HE) staining

The tissue specimens were sliced into 4 μm sections and mounted on silanized glass slides. After deparaffinization and hydration, the sections were stained by incubation with haematoxylin solution at 35°C for 3 minutes. Subsequently, the sections were immersed 5 times in 0.5% acid ethanol (1% HCl in 70% ethanol) and rinsed in distilled water. Then, the sections were stained by incubation with eosin solution at 35°C for 1 minutes, dehydrated with graded alcohol and cleared with xylene. Finally, the sections were examined under a light microscope (IX81, Olympus) at 20× magnification.

### 2.13 Co‐immunoprecipitation (Co‐IP) and mass spectrometric (MS) analysis

3 × Flag‐Vector and 3×Flag‐TAK1 MKN45 cells were resuspended with an appropriate amount of weak lysate (cat. no. P0013D; Beyotime) and placed on ice for lysis for 20 minutes after the cells were collected. After collecting the supernatant by centrifugation, the protein sample concentration was quantified using a BCA kit (cat. no. P0009; Beyotime). TAK1 inhibitor 5Z‐7‐oxozeaenol was purchased from Sigma‐Aldrich (cat. no. O9890; Sigma‐Aldrich). The Co‐IP assay was performed by protein A/G plus agarose (cat. no. sc‐2003; Santa Cruz) experiment. One microgram of 3 × Flag primary antibody (cat. no. 20543‐1‐AP; Proteintech) was added to the samples and stirred at 4°C for 5 hours. Then, the samples were administered with 20 µL of protein G and placed in a rotary mixer for 1 hour at room temperature. Subsequently, the samples were centrifuged, protein A/G plus was collected and the agarose beads were washed with weak lysate three times, each time with 1 mL of lysis solution for 10 minutes. After washing the agarose beads, the samples were centrifuged, the supernatant was removed, and an appropriate amount of 1× loading buffer was added. All samples were then placed in a 100°C water bath for 10 minutes. Finally, the obtained supernatant was used for subsequent Western blot experiments. The immunoprecipitates were sent to Applied Shanghai Protein Technology Co. Ltd. The analysis was conducted on a Q Exactive mass spectrometer coupled to Easy nLC (Thermo Fisher Scientific) using a routine method.

### 2.14 Immunofluorescence

To detect the TAK1 and YAP cell location, the GCSCs were incubated in 4% paraformaldehyde solution for 15 minutes and permeabilized in 0.5% Triton at room temperature for 5 minutes. A 5% BSA solution was used to block the samples, which were subsequently incubated with anti‐TAK1 (cat. no. sc‐7967, 1:500, Santa Cruz) and anti‐YAP (cat. no. 4912, 1:200, CST) for 30 minutes. Then, the samples were washed three times and incubated with secondary antibody for 30 minutes. Finally, the cells were DAPI‐stained and visualized using a microscope (IX81, Olympus).

### 2.15 Luciferase reporter assay

Luciferase reporter assays were conducted through the Dual‐luciferase Reporter Assay System (Promega). The wild‐type or mutant SOX2 that had the predicted binding site was settled and incorporated into a pGL3 dual‐luciferase vector to constitute the pGL3‐SOX2‐wild type (SOX2‐wt) or pGL3‐SOX2‐mutant (SOX2‐mut) reporter vector. The SOX2‐wt or SOX2‐mut co‐transfection was performed with shYAP or negative control into HEK293T cells using Lipofectamine 2000. Luciferase activity was measured according to the manufacturer's guidelines after 48 hours of transfection. In the same way, pGL3‐SOX9‐wild type (SOX9‐wt) and pGL3‐SOX9‐mutant (SOX9‐mut) reporter vectors were constructed, and co‐transfected with shYAP or negative control into HEK293T cells. The experiment was carried out in triplicate and expressed as the mean ± the standard deviation (SD).

### 2.16 Cell counting Kit‐8 (CCK‐8) assay

Cell Counting Kit‐8 (CCK‐8, Beyotime) assay was carried out according to the manufacturer's guidelines. After plating the cells with different treatments in 96‐well plates, each well was administered with the CCK‐8 reagent. The cellular feasibility determination was performed by obtaining the absorbance at the 450 nm wavelength through a microplate reader.

### 2.17 Statistical analysis

All in vivo and in vitro data presented were based on at least 3 independent experiments. Pearson chi‐square test and Student's *t* test were used for comparisons between two or multiple groups, respectively. Dunnett's *t* test was used to test the difference between the experimental and the control group sequentially. Survival analysis was conducted using Kaplan‐Meier method. The differences were considered statistically significant when the *P* < .05. SPSS 21.0 and Prism 8.0 software were utilized for data analysis.

## 3 RESULTS

### 3.1 TAK1 is up‐regulated in gastric cancer

**FIGURE 1:**
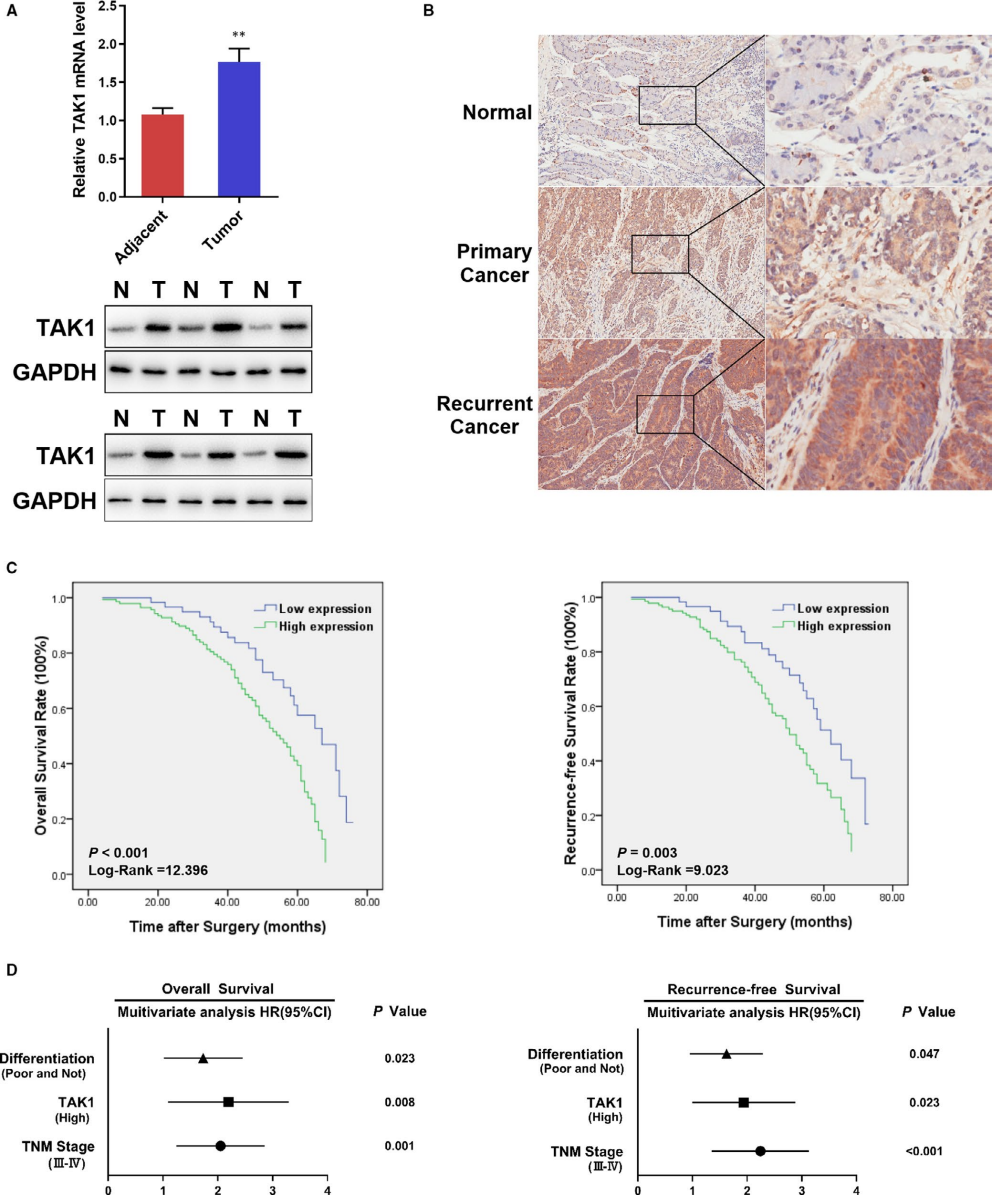
Up‐regulation of TAK1 in human gastric tissues. A, The mRNA expression levels of TAK1 in GC and adjacent tissues (**P < .01); the protein expression levels of TAK1 in 6 paired GC(T) and adjacent tissues(N) were evaluated by Western blot. B, Representative IHC staining images of TAK1 in normal, primary cancer and recurrent cancer tissues of GC. C, The OS (left panel) and RFS (right panel) for the high and low TAK1 expression groups according to IHC staining intensity were analysed by Kaplan‐Meier analysis. D, A multivariate analysis by the Cox multivariate proportional hazard regression model indicated that up‐regulation of TAK1 may be an independent prognostic factor for the OS and RFS rates in patients with GC. The HRs are presented as the means (95% CI)

To study the TAK1 potential role in GC, the TAK1 expression level was measured in GC and the related adjacent non‐cancerous samples. As shown in Figure 1A, the TAK1 protein and mRNA levels in GC tissue were significantly increased compared to adjacent normal tissues. Protein expression level was also detected by immunohistochemistry (IHC) staining of 200 pairs of GC and related adjacent non‐cancerous samples. The results showed that IHC scores of TAK1 expression in tumour tissues were significantly enhanced compared to normal tissues. Remarkably, the TAK1 expression level has been found to be significantly higher in recurrent GC than in primary GC (Figure 1B). To understand the relationship between TAK1 expression and clinicopathological parameters of GC patients, gender, age, drink, tumour location, differentiation, and T, N and TNM stages were analysed. An increased TAK1 expression was significantly associated with the poor and not tumour differentiation (*P* = .006), advanced N stage (*P* = .033) and advanced TNM stage (*P* = .016) (Table 1), but it was not significantly associated with gender, age, drink, tumour location and T stage.

**TABLE 1:** Relationships between TAK1 protein expressions (immunohistochemical staining) in gastric cancer and various clinicopathological variables

Variables	Total	TAK1 expression		χ^2^	*P*
		Low (n = 59)	High (n = 141)		
Gender					
Female	65	20	45	0.075	0.785
Male	135	39	96		
Age (years)					
≤60	75	24	51	0.361	0.548
>60	125	35	90		
Drink					
Yes	117	39	78	1.992	0.158
No	83	20	63		
Tumour location					
Antrum	144	39	105	1.444	0.229
Other	56	20	36		
Differentiation					
Well/moderate	109	41	68	7.585	0.006
Poor/not	91	18	73		
T stage					
T1/T2	82	26	56	0.326	0.568
T3/T4	118	33	85		
N stage					
N0/N1	92	34	58	4.555	0.033
N2/N3	108	25	83		
TNM stage					
I/II	120	43	77	5.786	0.016
III/IV	80	16	64		

Abbreviations: M, Metastasis; N, Node; T, Tumour; TAK1, TGF‐β‐activated kinase 1; TNM, Tumour Node Metastasis.

Patient follow‐up data were evaluated to further identify the contribution of TAK1 expression to the GC patient prognosis. The 200 GC patients were classified into two groups based on the TAK1 IHC scores of their tumours: a high TAK1 expression group (141/200) and a low TAK1 expression group (59/200). Higher TAK1 expression patients showed worse overall survival (OS; mean OS times were 51 vs. 42 months, respectively; log‐rank = 12.396, *P* < .001) and lower recurrence‐free survival (RFS; mean RFS times were 47 vs. 40 months, respectively; log‐rank = 9.023, *P* = .003; Figure 1C). Multivariate analysis indicated that the expression level of TAK1 may be an independent risk factor for OS and RFS in GC patients (Figure 1D). The high TAK1 expression group exhibited smaller OS and RFS rates (OS: hazard ratio (HR) = 2.011, 95% confidence interval (CI), 1.198‐3.376, *P* = .008; RFS: HR = 1.786, 95% CI, 1.083‐2.946, *P* = .023). These data suggest that the TAK1 expression level can be useful as an independent factor for GC prognosis prediction.

### 3.2 TAK1 has tumour‐promoting function in gastric cancer cells

**FIGURE 2:**
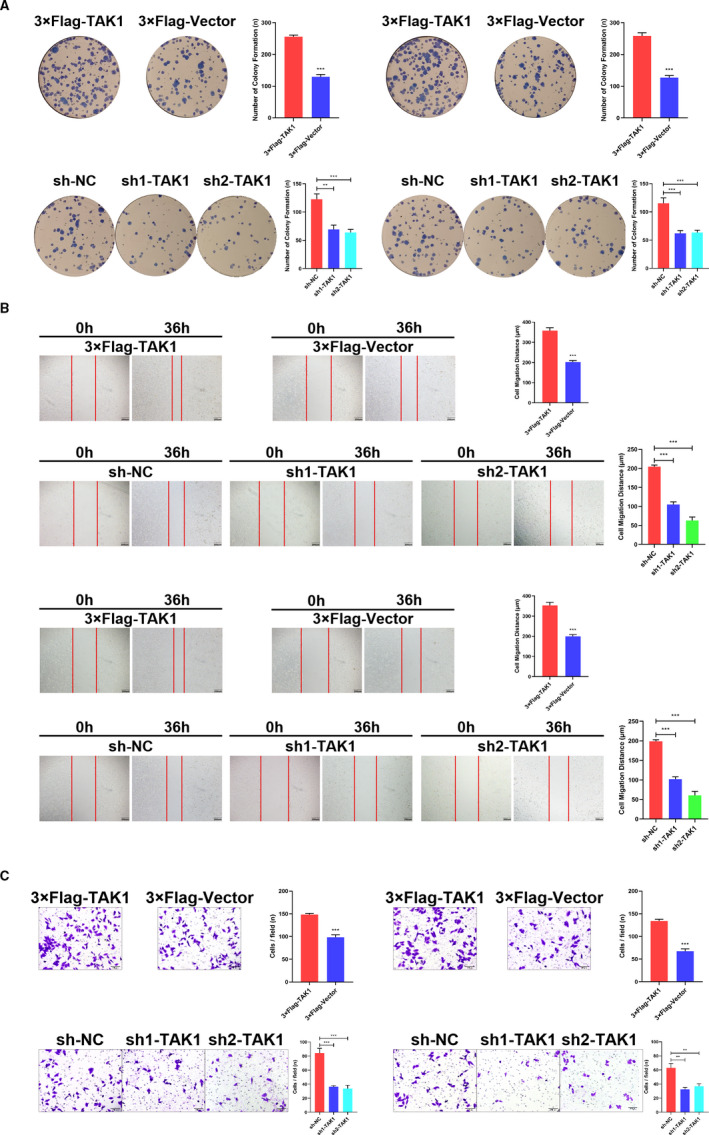
Tumour‐promotive effects of TAK1 in GC cells. A, Representative images of colony formation induced by 3 × Flag‐TAK1, 3 × Flag‐Vector, sh‐NC, sh1‐TAK1 and sh2‐TAK1 in MKN45 and MGC803 cell lines (left and right panel). The numbers of colonies were measured and are shown in the bar graph. All data were mean ± SD and from three independent experiments (**P < .01, ***P < .001). B, A cell wound‐healing assay showed that cell motility was promoted by overexpression of TAK1 and was decreased after TAK1 knockdown in the MKN45 and MGC803 cell lines (upper and lower panel). Microscopic images were acquired at 0 and 36 h (left panel; magnification, ×40). The migratory distance of the cells was measured and is shown in the bar graph. All data were mean ± SD and from three independent experiments (right panel; ***P < .001). C, Cell invasion assays of 3 × Flag‐TAK1, 3 × Flag‐Vector, sh‐NC, sh1‐TAK1 and sh2‐TAK1 in MKN45 and MGC803 cell lines (left and right panel). Invaded cells were fixed and stained with crystal violet (magnification, ×100). The number of invaded cells was calculated and is shown in the bar graph. All data were mean ± SD and from three independent experiments (**P < .01, ***P < .001)

Functional in vivo and in vitro analyses were realized to further understand the TAK1 role in GC. A noticeably higher TAK1 expression was observed in the GC cell lines MGC803, MKN45, AGS, MKN28 and HGC27, in comparison with the non‐cancerous gastric cell line GES‐1 (Figure S1A). A lentivirus encoding TAK1 was used to construct TAK1‐overexpressing MKN45 and MGC803 cells, which subsequently exhibited high TAK1 expression levels (Figure S1B,C). Moreover, two different short hairpin RNA (shRNA) sequences targeted against TAK1 was transfected into MKN45 and MGC803 cells, which identified low TAK1 protein expression levels (Figure S1B,C). Compared with control cells, the mRNA and protein expression level of TAK1 in TAK1‐overexpression cells was about 2 to 5 times higher. And expression of the shRNA specifically reduced the mRNA and protein expression of TAK1 by about 50%‐70%. Colony formation assays indicated that enhanced colony formation rates were detected in TAK1‐overexpressing MKN45 and MGC803 cell lines compared to the control cells. Besides, sh‐TAK1 significantly inhibited the colony formation frequency in both MKN45 and MGC803 cells compared to the control cells (Figure 2A). The TAK1 effect on invasion and metastasis of GC cells has also been evaluated. Wound healing assays and Matrigel invasion showed that TAK1 efficiently promoted the invasive and migratory abilities of MKN45 and MGC803 cell lines compared to the control cells. These abilities of MKN45 and MGC803 cell lines were significantly decreased when the TAK1 expression was disturbed (Figure 2B,C).

**FIGURE 3:**
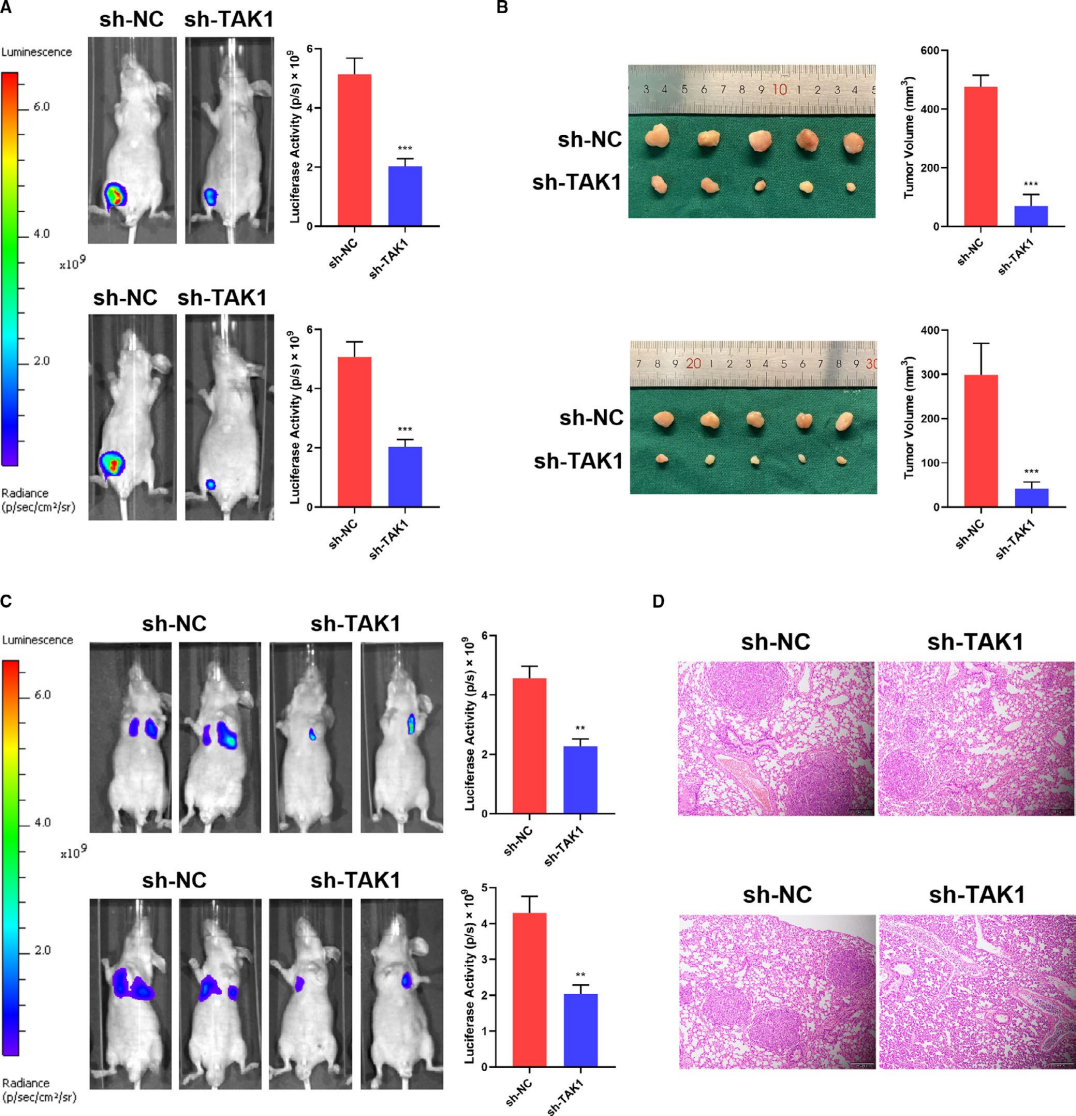
Tumour‐promotive effects of TAK1 in vivo tumorigenesis. A, Representative bioluminescent images are shown for tumours derived from nude mice induced by sh‐NC and sh‐TAK1 in MKN45 and MGC803 cell lines (upper and lower panel) after subcutaneous injection. The statistical analysis of luciferase activity is shown in the bar graph (n = 5 mice per group, ***P < .001). B, Representative images are shown for the tumours derived from nude mice induced by sh‐NC and sh‐TAK1 in MKN45 and MKC803 cell lines (upper and lower panel) after subcutaneous injection. The volume of tumour was measured and is shown in the bar graph (n = 5 mice per group, ***P < .001). Scale bars = 1 cm. C, Representative bioluminescent images are shown for tumours derived from nude mice induced by sh‐NC and sh‐TAK1 in MKN45 and MKC803 cell lines (upper and lower panel) after tailing intravenous injection. The statistical analysis of luciferase activity is shown in the bar graph (n = 6 mice per group, *P < .05, **P < .01). D, Representative haematoxylin and eosin (H&E) images of metastatic nodules from mouse lung tissue sections of the sh‐NC and sh‐TAK1 in MKN45 and MKC803 cell lines (upper and lower panel). Scale bars = 200 μm

For in vivo tumour formation evaluation, luciferase‐labelled MKN45‐shNC, MGC803‐shNC, MKN45‐sh‐TAK1 (sh1‐TAK1 sequence) and MGC803‐sh‐TAK1 (sh1‐TAK1 sequence) cells were subcutaneously inoculated in nude mice. Live imaging showed a notably luciferase activity reduction in low TAK1 expressing level tumours compared to the control groups (Figure 3A). Furthermore, the size and volume of the tumours induced by TAK1‐interfered GC cells were significantly reduced compared to those of the control cells (Figure 3B). A lung metastasis model was employed to further identify the TAK1 role in GC metastasis. This model was established by caudal vein administration in nude mice of luciferase‐labelled sh‐TAK1‐GC cells with related shNC cells used as a control. Tumour size and luciferase activity were significantly reduced in tumours induced by GC cells with TAK1 interference compared to the tumours induced in control cells (Figure 3C,D). These results reveal that TAK1 promotes the malignant GC cell phenotype both in vivo and in vitro.

### 3.3 TAK1 is expressed at high levels and promote self‐renewal in gastric cancer stem cells

**FIGURE 4:**
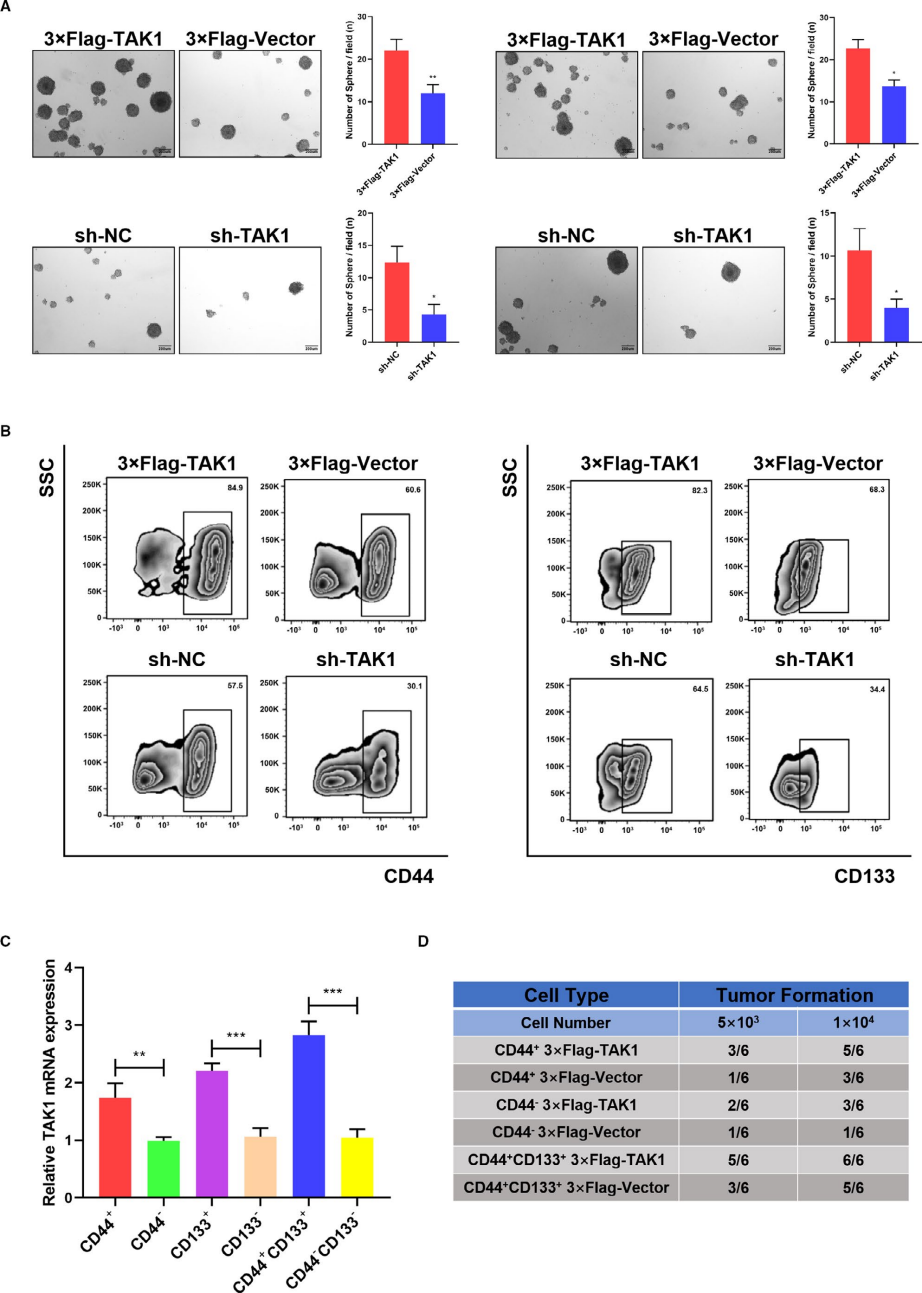
Expression of TAK1 in gastric CSCs. A, Representative images of sphere formation in 3 × Flag‐TAK1, 3 × Flag‐Vector, sh‐NC, sh‐TAK1 in MKN45 and MGC803 cell lines (left and right panel). The numbers of sphere formation were measured and are shown in the bar graph. All data were mean ± SD and from three independent experiments (*P < .05; **P < .01), scale bars = 200 μm. B, Flow cytometry analysis of CD44^+^ or CD133^+^ populations in spheres derived from 3 × Flag‐TAK1, 3 × Flag‐Vector, sh‐NC, sh‐TAK1 in MKN45 cells. Representative results from three independent experiments are shown. C, TAK1 mRNA expression in CD44^+^, CD133^+^ and CD44^+^CD133^+^ populations of MKN45 cells were detected by RT‐PCR (**P < .01; ***P < .001). Representative results from three independent experiments are shown. D, CD44^+^, CD44^−^ and CD44^+^CD133^+^ cells isolated from 3 × Flag‐TAK1, 3 × Flag‐Vector cells were inoculated into NOD‐SCID mice subcutaneously and the tumorigenicity was evaluated two months after inoculation (n = 6 mice per group)

The CSC presence figures prominently in tumour proliferation, invasion and recurrence, resulting in poor outcomes and limited therapeutic options. Given the TAK1 role in promoting various GC malignant phenotypes, the TAK1 expression level has been estimated in GCSCs. As self‐renewal is a CSCs distinct feature, GCSCs were enriched by inducing the formation of GC spheroids. As shown in Figure 4A, an enhanced GC spheroid formation was observed in TAK1‐overexpressing GC cells. On the other hand, TAK1 inhibition attenuated the tumour spheroid‐formation frequencies in MKN45 and MGC803 cells. Flow cytometry analysis revealed that the CD44^+^ and CD133^+^ GC cell proportion was increased in TAK1‐overexpressing GC spheroids and decreased in TAK1‐knockdown spheroids (Figure 4B).

Therefore, we believed that the cancer stem cell population was enriched by GC spheroid formation. To determine the TAK1 expression profile in GCSCs, the TAK1 mRNA expression level was compared between CD44^+^, CD133^+^ or CD44^+^ CD133^+^ GC cells and CD44^−^, CD133^−^ or CD44^−^ CD133^−^ GC cells which were obtained by flow cytometry sorting. RT‐qPCR analysis showed a significantly increased TAK1 mRNA expression in CD44^+^ or CD133^+^ GC cells compared to CD44^−^ or CD133^−^ GC cells. Consistently, a markedly higher TAK1 mRNA expression was observed in CD44^+^ CD133^+^ GC cells than in CD44^−^ CD133^−^ GC cells (Figure 4C). In vivo xenograft tumour formation experiments revealed that the tumorigenic ability of TAK1‐overexpressing CD44^+^ CD133^+^ GC cells was significantly greater than that of CD44^+^ and/or CD44‐GC cells alone (Figure 4D). These results indicated that TAK1 is overexpressed in GCSCs.

### 3.4 TAK1 stabilizes YAP independently from its kinase activity

**FIGURE 5:**
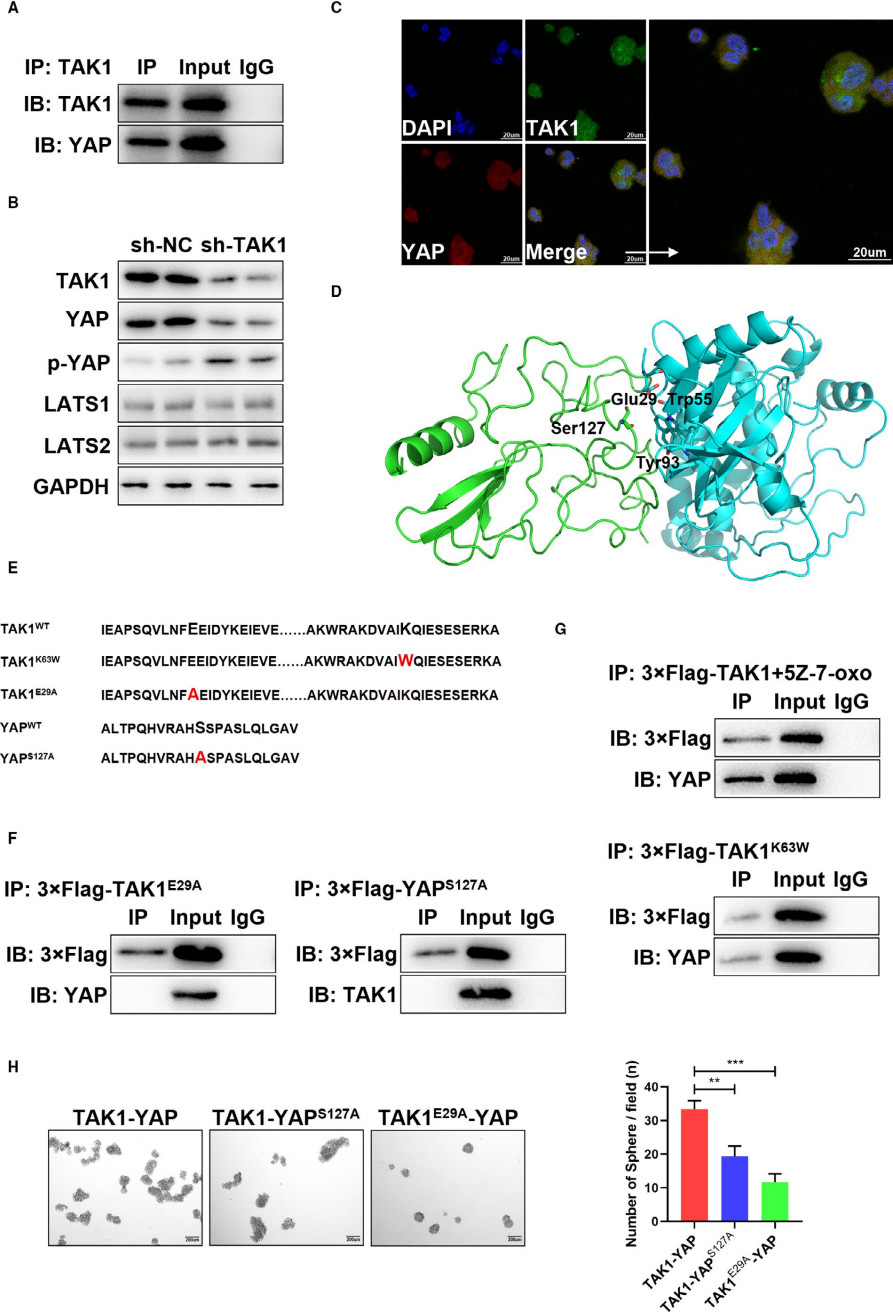
The binding of TAK1 and YAP in GCSCs. A, TAK1 interacts with YAP, as evidenced by a Co‐IP assay in MKN45‐derived gastric cancer spheroids. Immunoprecipitations were performed using anti‐TAK1 antibodies to identify indicator protein expression. B, Representative images showing the expression levels of YAP, p‐YAP, LATS1 and LATS2 after TAK1 knockdown in MKN45‐derived gastric cancer spheroids. C, Representative double immunofluorescence staining images showing that co‐localization of TAK1 (green) and YAP (red) proteins in MKN45‐derived gastric cancer spheroids (upper and lower panel). D, The site in TAK1 that can form a hydrophobic surface near residue Ser127 in YAP was predicted by Hdock server and was visualized by LigPlot and PyMOL. E, The design of mutation sites for TAK1^K63W^, TAK1^E29A^ and YAP^S127A^, compared with wild type of TAK1 and YAP. F, TAK1^E29A^ does not interact with YAP, and YAP^S127A^ also does not interact with TAK1 through Co‐IP assay in MKN45‐derived gastric cancer spheroids. G, After using the TAK1 kinase inhibitor 5Z‐7‐xox, TAK1 does not interact with YAP and TAK1^K63W^ (a mutation in the TAK1 kinase site) also does not interact with YAP through Co‐IP assay in MKN45‐derived gastric cancer spheroids. H, Representative images of sphere formation in TAK1, YAP^S127A^ and TAK1^E29A^ in MKN45 cells. The numbers of sphere formation were measured and are shown in the bar graph. All data were mean ± SD and from three independent experiments (**P < .01; ***P < .001), scale bars = 200 μm

To further dissect the mechanism by which TAK1 contributes to self‐renewal of GCSCs, we performed the liquid chromatography tandem mass spectrometry (LC‐MS/MS) to qualitatively analyse components of TAK1 binding protein in GCSCs. LC‐MS/MS analysis identified that TAK1 interacted with YAP, which inhibited by Hippo pathway (Figure S2A). As there are no previous reports that TAK1 can interact with YAP and regulate biological processes in CSCs, immunoprecipitation was performed to attest the TAK1 binding to YAP (Figure 5A). Further experiments showed that the TAK1 loss was associated with declines in YAP levels and negatively related to p‐YAP. Other molecules of the HIPPO pathway were not shown to be related to TAK1 (Figure 5B). Moreover, immunofluorescence (IF) analysis proved that there is a direct interaction between YAP and TAK1 in GCSCs (Figure 5C).

The Hippo signalling pathway kinase cascade partially inhibits YAP by phosphorylation of its Ser127 residue, resulting in binding of YAP 14‐3‐3 and cytoplasmic retention.^32^ To understand how TAK1 and YAP interact, the way of binding between TAK1 and YAP was predicted by protein‐protein docking simulations. As shown in Figure 5D, the TAK1 residues Glu29, Trp55 and Tyr93 form a hydrophobic surface near to the Ser127 residue in YAP. More precisely, it is the Glu29 residue of TAK1 that establishes hydrophobic interactions with the Ser127 residue of YAP. TAK1 residues Trp55 and Tyr93 establish hydrophobic interactions with YAP Ser128 residue. Co‐IP assays performed TAK1‐mutated at Glu29 site and YAP‐mutated at Ser127 site confirmed the docking simulation results in GCSCs (Figure 5E,F).

MKN45 cells were administered with the TAK1 kinase inhibitor 5Z‐7‐oxozeaenol^33^ and with the inactive form of TAK1 kinase, in which the Lys63 residue of the ATP‐binding site is substituted by a Trp residue. The results showed that the combination of YAP and TAK1 has no significant correlation with the TAK1 kinase activity in GCSCs (Figure 5G). In addition, spheroid‐formation assays revealed that the TAK1 and YAP combination promotes GCSCs self‐renewal (Figure 5H). These results indicate that YAP can avoid being degraded in the cytoplasm by covering its phosphorylation sites with TAK1 and induces an increase in non‐phosphorylation YAP, favours YAP nuclear localization and promotes YAP target genes in GCSCs.

### 3.5 TAK1/YAP axis activates SOX2 and SOX9 transcription in the gastric cancer stem cells self‐renewal

**FIGURE 6:**
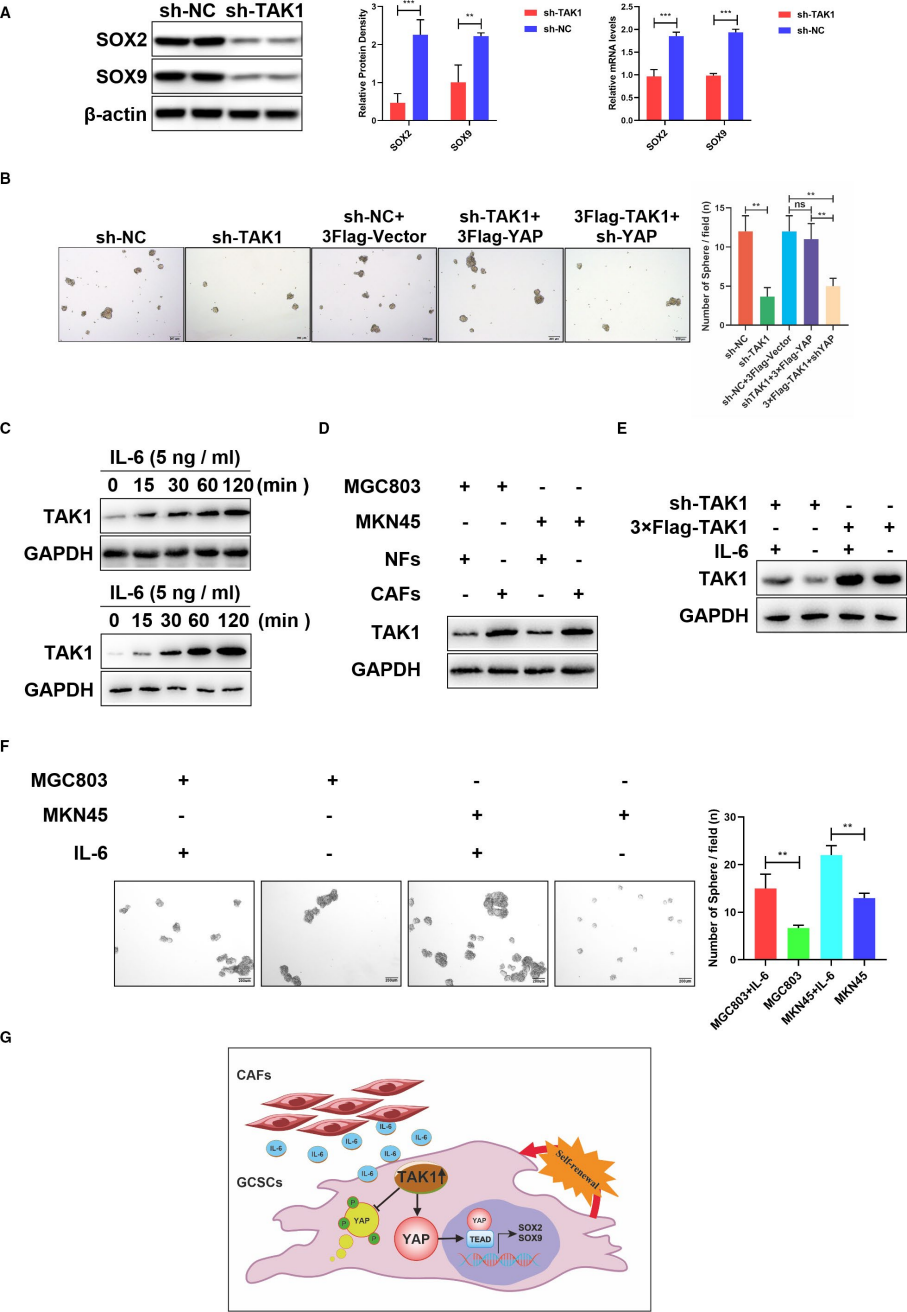
IL‐6‐dependent TAK1/YAP promotes the sphere formation in GC cells. A, Representative images showing the protein and mRNA expression levels of SOX2 and SOX9 after TAK1 knockdown in MKN45‐derived gastric cancer spheroids. The quantification of bands represented in a bar graph (**P < .01, ***P < .001). B, Representative images of sphere formation in sh‐NC, sh‐TAK1, sh‐NC + 3Flag‐Vector, sh‐TAK1 + 3Flag‐YAP and 3Flag‐TAK1 + shYAP in MKN45 cells. The numbers of sphere formation were measured and are shown in the bar graph. All data were mean ± SD and from three independent experiments (**P < .01), scale bars = 200 μm. C, Representative images showing the expression levels of TAK1 increased with the incubation time of IL‐6 in MKN45 and MGC803 cell lines (upper and lower panel). D, Representative images showing the expression levels of TAK1 increased when co‐cultured with CAFs, compared to co‐cultured with NFs in MKN45 and MGC803 cell lines. E, The representative expression levels of TAK1 with the incubation of IL‐6 in sh‐TAK1 and 3×Flag‐TAK1 MKN45 cell lines. F, Representative images of sphere formation in MKN45 and MGC803 cell lines with or without IL‐6 incubation. The numbers of sphere formation were measured and are shown in the bar graph. All data were mean ± SD and from three independent experiments (**P < .01), scale bars = 200 μm. (g). IL‐6 is secreted by CAFs in the cell microenvironment and promotes the expression of TAK1 in cells. TAK1 prevents the degradation of YAP in the cytoplasm by binding to YAP and promotes the transcription of SOX2 and SOX9 in the nucleus. Finally, TAK1 promoted the self‐renewal ability of gastric cancer stem cells

Yes‐associated protein exerts its role as a transcriptional coactivator mainly through interaction with the transcription factor TEAD1.^34^ Then, the UCSC^35^ and JASPAR databases^36^ were used to examine the promoter region of several stemness‐associated genes for TEAD1 binding sites (Figure S2B). The competence for TEAD1 binding was assessed by a luciferase reporter system as SOX2 and SOX9 have been described as a relevant regulator of the population of GCSCs.^37^, ^38^ The dual‐luciferase results show that shYAP reduced the SOX2 and SOX9 promoter activity (Figure S2C). In addition, decreased SOX2 and SOX9 expression has been shown to be related to TAK1 interfered expression (Figure 6A).

To study the role of TAK1/YAP axis in GC progression, it was investigated whether the TAK1/YAP axis regulates the GCSCs self‐renewal. The TAK1 knockdown decreased the GCSCs sphere formation ability. However, the sh‐TAK1 inhibitory effect on GCSCs self‐renewal was recovered by YAP overexpression. Furthermore, the GCSC spheroid‐forming ability was not increased after YAP was interfered in the overexpressing TAK1 cell line (Figure 6B). Flow cytometry analysis showed similar results (Figure S3A). These results confirm that TAK1/YAP axis participates in the GCSCs self‐renewal, thereby promoting the tumour growth in GC.

### 3.6 IL‐6 promotes increased TAK1 expression in gastric cancer

Cancer stem cells are regulated by the tumour microenvironment, and some cytokines in the malignant tumour are closely related to the self‐renewal, maintenance and growth of these cells.^39^ IL‐6 has been suggested to enhance the TAK1 expression level and induce the association between TAK1 and GNAI3.^40^ Thus, TAK1 expression level was detected in IL‐6‐treated MKN45 and MGC803 cells to verify whether the TAK1 expression in GC is induced by IL‐6. The results showed that the TAK1 expression levels increased with increasing IL‐6 incubation time (Figure 6C). In addition, the TAK1 expression levels in cancer cells that were co‐cultured with CAFs were higher than those co‐cultured with NFs (Figure 6D). Similarly, we observed that IL‐6 can enhance the expression of TAK1 at different expression levels of TAK1 (Figure 6E). GCSCs have been enriched by inducing spheroid formation through co‐cultured cells. As shown in Figure 6F, the tumour spheroid‐formation frequencies are enhanced in the culture environment in which IL‐6 was added. Flow cytometry analysis showed that the CD44^+^ and CD133^+^ GC cell proportion was increased in GC spheroids when co‐cultured with IL‐6 (Figure S3B). These results reveal that increased TAK1 expression in GC cells can be regulated by the increase in the IL‐6 levels.

### 3.7 TAK1 inhibits the chemosensitivity of gastric cancer cells

**FIGURE 7:**
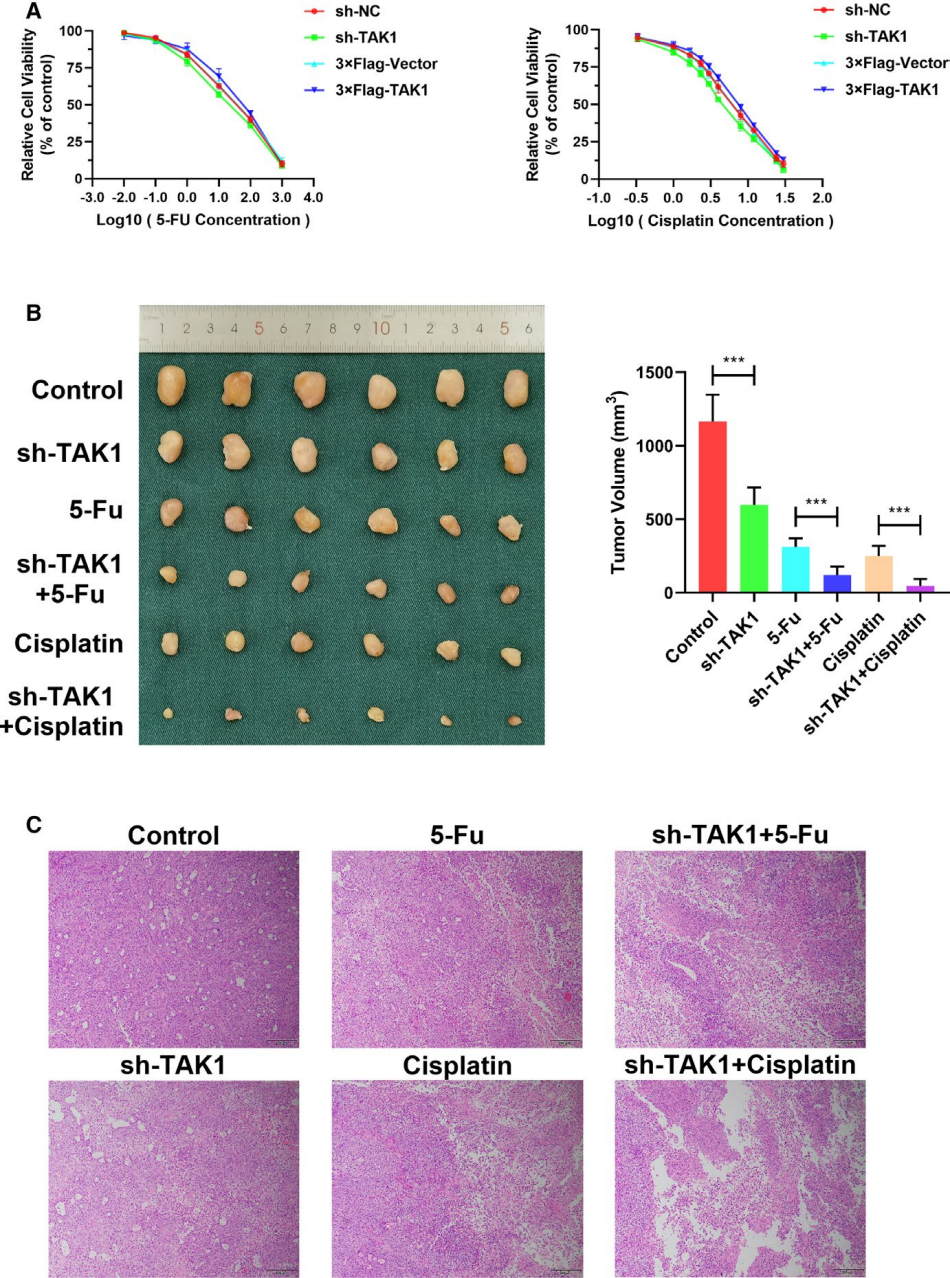
TAK1 inhibits the chemosensitivity of GC cells. A, CCK‐8 assay was used to compare cell viability in MKN45 cells with TAK1 overexpression and knockdown after treatment with 5‐Fu and cisplatin, respectively (left and right panel). Data are means ± SD of three independent experiments. B, Representative image of xenograft tumorigenesis and treatment with TAK1, 5‐Fu and cisplatin in nude mice. A 1 mm^3^ piece of the xenograft was subcutaneously implanted in a 3‐ to 4‐week‐old male nude mouse, individually. When the tumour reached approximately 5 mm in diameter, the nude mice were divided into six groups (n = 6 per group). Representative images of xenograft tumours in nude mice after different treatments (left panel). The tumour volume of each group of mice is summarized (right panel; *P < .05, **P < .01, ***P < .01). C, Xenografts were fixed and embedded in paraffin and stained with H&E for analysis of tumour necrosis induced by TAK1/chemotherapeutics (scale bars = 200 μm)

As TAK1 was identified as a GCSCs self‐renewal promoter, it was subsequently investigated whether TAK1 can affect the GC cell resistance to chemotherapeutic drugs 5‐fluorouracil (5‐FU) and cisplatin. The cell viability was significantly increased in TAK1‐overexpressing MKN45 cells, as evidenced by CCK‐8 analysis results. The 5‐Fu IC50 value was increased from 165.5 to 363.6 and the cisplatin IC50 value was increased from 7.752 to 8.444 in TAK1‐overexpressing MKN45 cell, in comparison with the control cells. In contrast, the 5‐Fu IC50 value was decreased from 201.3 to 113.6 and the cisplatin IC50 value was decreased from 7.437 to 5.670 in TAK1‐interfered MKN45 cells, compared to the control cells (Figure 7A). In order to evaluate whether TAK1 could increase the chemoresistance of GC cell in vivo, a xenograft tumour model was established, which were arbitrarily separated into six groups and administered with an empty vector (control), sh‐TAK1, 5‐FU, sh‐TAK1 + 5‐Fu, cisplatin and sh‐TAK1+ cisplatin, respectively. The tumour size and volume were significantly reduced in the sh‐TAK1, 5‐FU and cisplatin treatment groups in comparison with the control group. Remarkably, the combined treatments of sh‐TAK1 and 5‐FU or cisplatin had a more effective inhibitory effect on tumour size and volume than any individual treatment (Figure 7B). Histological analysis showed a noticeably larger necrosis extension in the combined sh‐TAK1/chemotherapy treatments, compared to any individual treatment. These data reveal that sh‐TAK1 attenuated tumour growth in vivo and increased the GC cell chemosensitivity to 5‐FU and cisplatin (Figure 7C).

## 4 DISCUSSION

TAK1 is a member of the serine/threonine family of protein kinases. TAK1 mediates signalling transduction induced by TGF‐β and morphogenetic protein (BMP). Besides, it is involved in several cell functions, such as transcription regulation and apoptosis.^11^ Deregulation of all these processes is the basis of the hallmark cancer features, and regulated or deregulated TAK1 function mediates key roles in various tumours. Accumulated literature reports have proved that TAK1 participates in the initiation and development of several tumours.^41^ Yang and colleagues reported for the first time that the TAK1 expression level was increased in GC tissues and poor prognosis‐related in GC patients.^21^ However, the TAK1 role in the occurrence and development of GC is still poorly understood. In the present study, we confirmed once again that TAK1 is up‐regulated in GC tissue samples and found a positive correlation between TAK1 expression and various malignant features, especially tumour differentiation and poor prognosis of GC patients. We also observed ectopic expression of malignant phenotypes in TAK1 up‐regulated GC cells in vivo and in vitro. To date, this study is the first comprehensive evaluation of the TAK1 role in GC progression.

The presence of CSCs has been demonstrated for the first time in human acute myeloid leukaemia as a CD34^+^CD38^−^ population.^4^ So far, its presence has been confirmed in a variety of primary tumours, such as breast, liver, head and neck, colon, prostate, brain, pancreatic, lung, cervical and skin.^4^, ^5^, ^6^, ^7^ Several studies reporting the CSC presence in GC have emerged in recent years. Moreover, CD133 and CD44 are widely used as surface markers for GCSCs. In the current study, the GCSCs were sorted from the GC cells and identified as containing CD133 and CD44. Our data also showed that the CD44^+^ and CD133^+^ cell proportion in GCSCs is significantly increased and that the TAK1 mRNA expression level is positively correlated with this.

Transcriptional regulators YAP and TAZ have been reported as central malignancy determinants, due to their essential functions in tumour initiation, development, metastasis and chemoresistance.^23^ In addition, they play a crucial role in tumorigenesis through promotion of CSC features, such as self‐renewal, EMT, metastatic potential and chemoresistance.^42^, ^43^, ^44^ Previous reports revealed that YAP is stabilized by binding to TAK1, thereby activating the cell cycling of bone marrow–derived mesenchymal stem cells.^20^ Our results suggested that TAK1 binds to YAP and can promote the self‐renewal of GCSCs, thereby promoting the GC progress.

We treated gastric cells with 5Z‐7‐oxozeaenol, a TAK1 kinase inhibitor, and with the TAK1 kinase inactive form and found that the YAP and TAK1 binding has no significant correlation with the TAK1 kinase activity in GCSCs. Molecular docking simulations and Co‐IP assays showed that the TAK1 Glu29 residue can bind to the YAP Ser127 residue, thus occupying at least partially the site where YAP is phosphorylated by upstream LATS1/2 and eventually induce the nuclear translocation of YAP in GCSCs. This study is the first to present a molecular three‐dimensional model of TAK1 and YAP coupling in GCSCs.

TAK1 has been initially recognized as a kinase activated only via TGF‐β.^45^, ^46^ However, recent reports have shown that TAK1 can also be stimulated by several cytokines, including interleukins,^47^ FGFR1,^48^ FGFR3,^49^ G‐CSF and chemotactic factors.^50^ A previous study reported that IL‐6 can enhance TAK1 expression and induce the association between TAK1 and GNAI3.^40^ Then, it is not surprising that activation of TAK1 can be induced by IL‐6, as identified in the present work. What is more, multiple studies have shown that IL‐6 or its downstream signalling is capable of expanding CSCs in cancers of the breast, colon, ovary, lung, brain, and head and neck.^51^, ^52^, ^53^ Our experiments showed that IL‐6‐induced TAK1 activation is a relevant factor pointing to TAK1 as a central molecule for the GCSCs self‐renewal.

CSCs are highly resistant to chemotherapy and radiotherapy and, consequently, tend to co‐operate with cancer recurrence.^54^ There is evidence indicating that the TAK1 expression down‐regulation can reduce the protein levels of YAP/TAZ in vitro and in vivo and modulate the pancreatic cancer intrinsic chemoresistance.^55^ Then, we assessed the TAK1 potential therapeutic value in GC treatment. Both in *vivo* and in vitro experiments suggested that regulating TAK1 expression in GC has a remarkable therapeutic potential for the GC treatment, particularly with regard to tumour cell chemosensitivity to chemotherapy.

## 5 CONCLUSION

In summary, the results presented here show that TAK1 promotes tumour propagation, can be activated by IL‐6 and binds to YAP in GCSCs. Our results reveal a new critical paradigm in determining the GCSCs fate, which is a therapeutic promise for the GC treatment (Figure 6G).

## CONFLICT OF INTEREST

The authors declare that they have no conflict of interest.

## AUTHOR CONTRIBUTION

**Gang Wang:** Conceptualization (equal); Data curation (equal); Formal analysis (lead); Project administration (equal); Writing‐original draft (lead); Writing‐review & editing (lead). **Qikai Sun:** Conceptualization (equal); Data curation (equal); Formal analysis (equal); Project administration (equal); Software (equal); Visualization (equal); Writing‐original draft (equal); Writing‐review & editing (equal). **Hai Zhu:** Conceptualization (equal); Data curation (equal); Visualization (equal); Writing‐original draft (equal); Writing‐review & editing (equal). **Yihui Bi:** Software (equal); Visualization (equal). **Haixing Zhu:** Formal analysis (equal); Software (equal); Supervision (equal). **Aman Xu:** Conceptualization (lead); Data curation (lead); Funding acquisition (lead); Project administration (lead).

## 6 ETHICAL APPROVAL STATEMENT

All procedures followed were in accordance with the ethical standards of the responsible committee on human experimentation (The Fourth Affiliated Hospital of Anhui Medical University, China, certification no. 20150232) and with the Helsinki Declaration of 1964 and later versions. Informed consent to be included in the study, or the equivalent, was obtained from all patients. All institutional and national guidelines for the care and use of laboratory animals were followed (The Fourth Affiliated Hospital of Anhui Medical University, China, certification no. LLSC20150234).

## CONSENT FOR PUBLICATION

Not applicable.

## Supporting information

Fig S1

Fig S2

Fig S3

Table S1‐S2

## Data Availability

The data sets used and/or analysed during the present study are available from the corresponding author on reasonable request.
