# Supplementary figures and images for: The stabilization of yes‐associated protein by TGFβ‐activated kinase 1 regulates the self‐renewal and oncogenesis of gastric cancer stem cells
Source: J Cell Mol Med. 2021 Jun 1;25(14):6584–601. doi: 10.1111/jcmm.16660 (PMC8278074; doi:10.1111/jcmm.16660)

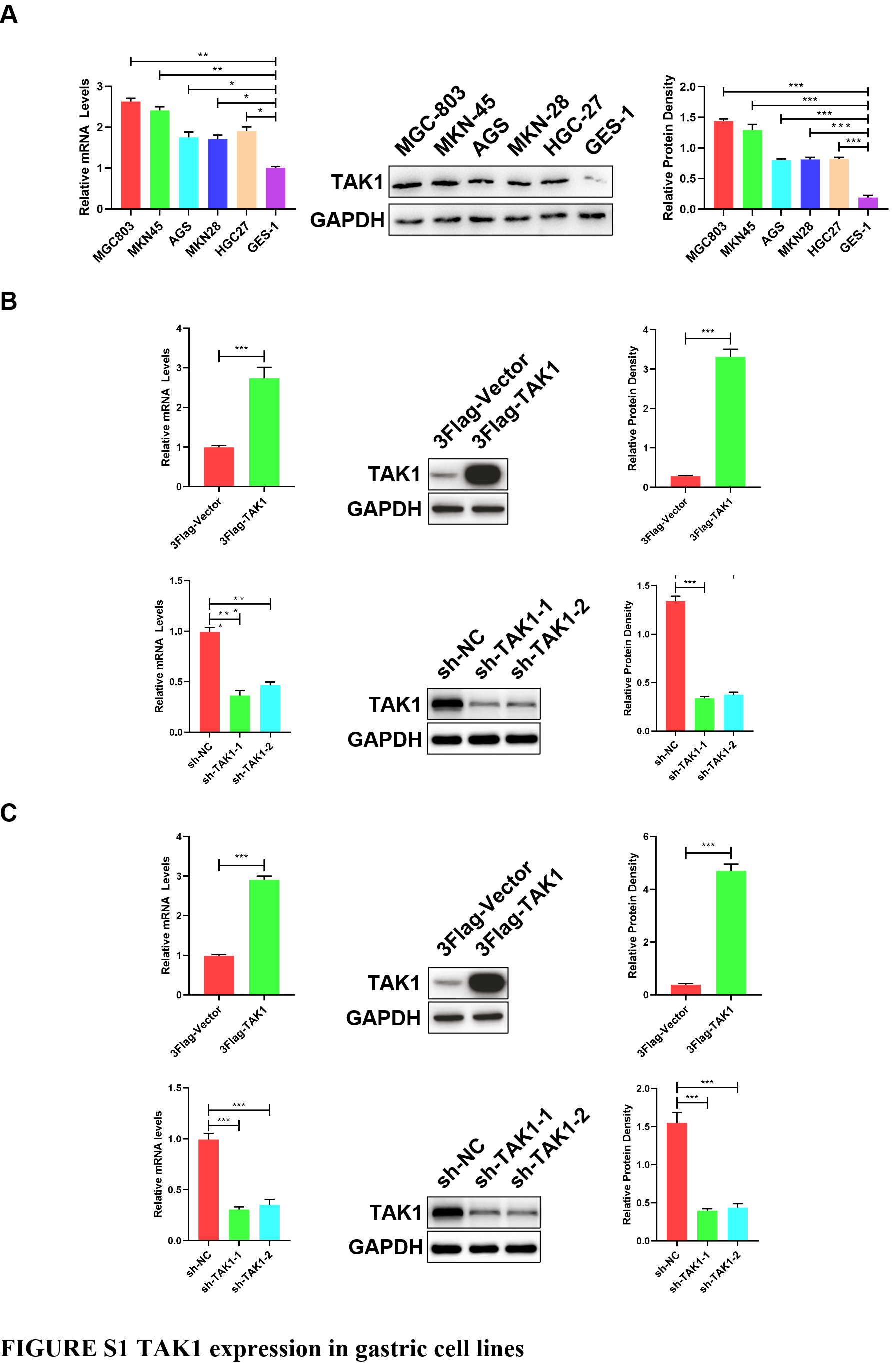

Supplement: Supplementary file 1 — Fig S1 [file JCMM-25-6584-s003.tif]

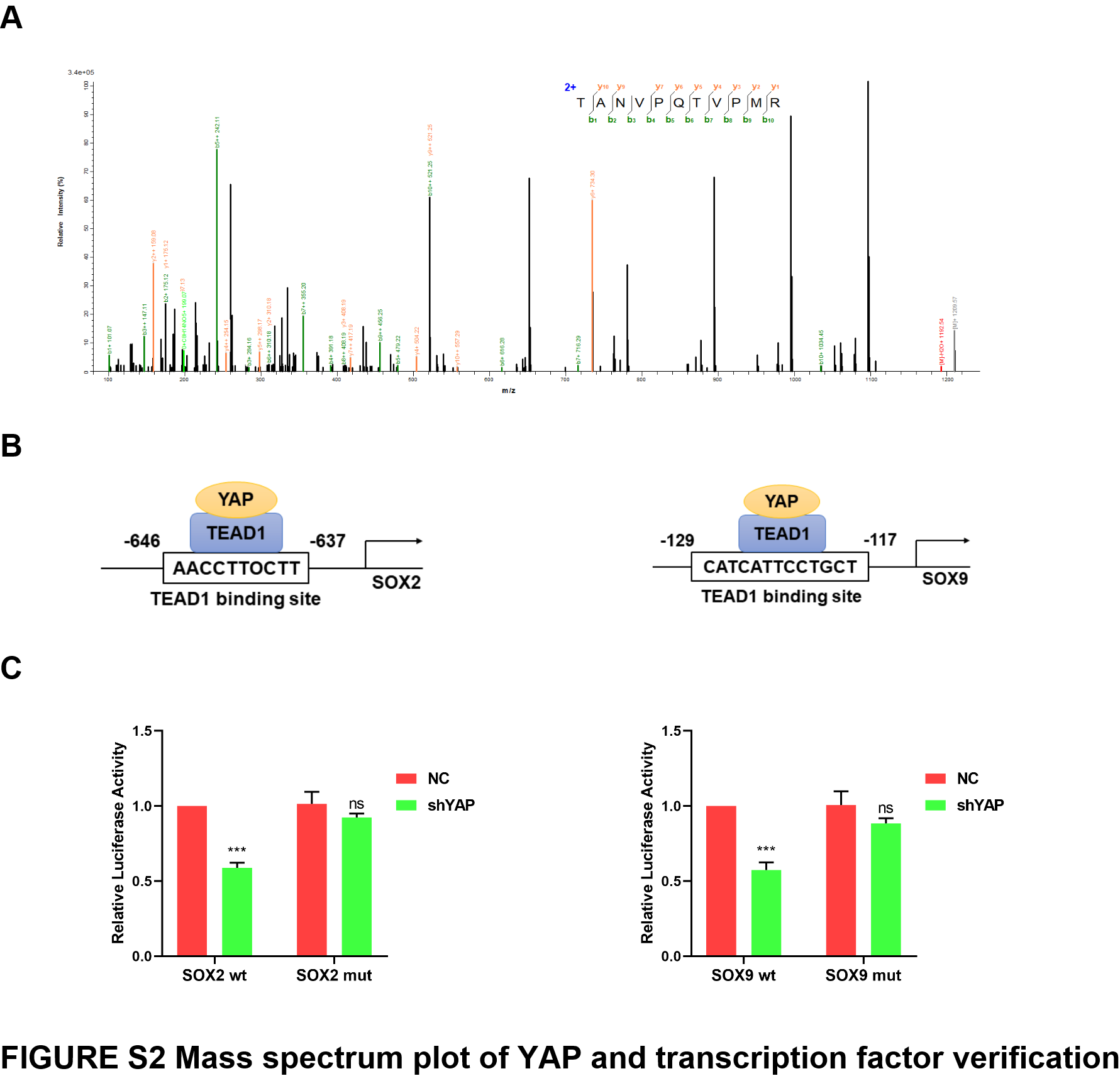

Supplement: Supplementary file 2 — Fig S2 [file JCMM-25-6584-s002.tif]

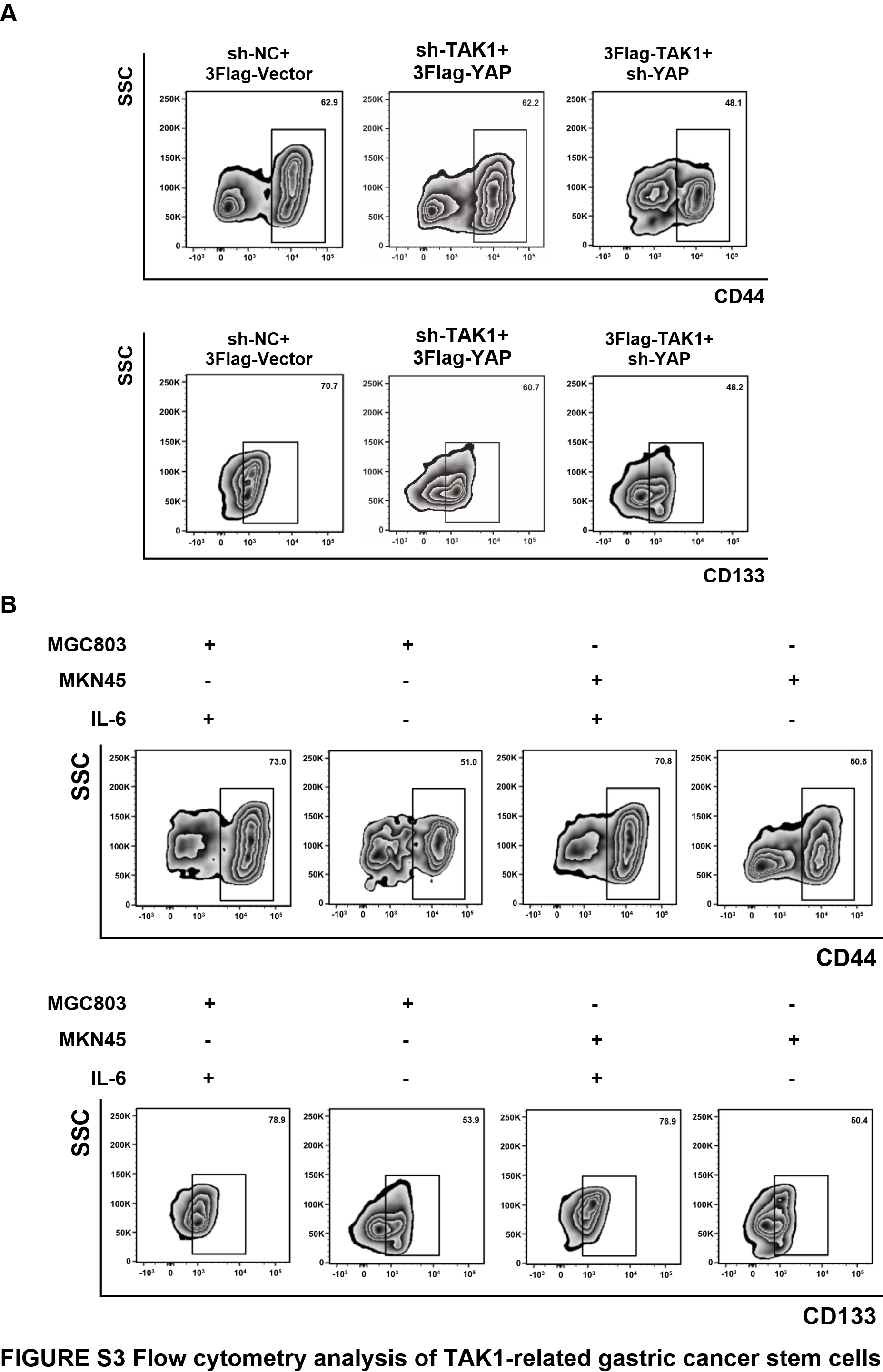

Supplement: Supplementary file 3 — Fig S3 [file JCMM-25-6584-s004.tif]
