# Supplementary material for: The stabilization of yes‐associated protein by TGFβ‐activated kinase 1 regulates the self‐renewal and oncogenesis of gastric cancer stem cells
Source: J Cell Mol Med. 2021 Jun 1;25(14):6584–601. doi: 10.1111/jcmm.16660 (PMC8278074; doi:10.1111/jcmm.16660)
Supplement: Supplementary file 4 — Table S1‐S2 [file JCMM-25-6584-s001.docx]

**Table S1. The target sequence of sh1-TAK1, sh2-TAK1, sh-NC and sh-YAP.**

| sh1-TAK1 | 5′-GAGGAAAGCGTTTATTGTATT-3′ |
| --- | --- |
| sh2-TAK1 | 5′-CCCAATGGCTTATCTTACATT-3′ |
| sh-NC | 5′-TTCTCCGAACGTGCACGTTTC-3′ |
| sh-YAP | 5′-GGAATTGAGAACAATGACGAC-3′ |

**Table S2. Sequences of specific primers for Quantitative real-time PCR (qPCR).**

| TAK1 | Forward | 5′-ACTCACTTGATGCGGT-3′ |
| --- | --- | --- |
|  | Reverse | 5′-CGGCGATCCTAGCTTC-3′ |
| GAPDH | Forward | 5′-AGGTCGGTGTGAACGGATTTG-3′ |
|  | Reverse | 5′-GGGGTCGTTGATGGCAACA-3′ |
| SOX2 | Forward | 5′-TGGACAGTTACGCGCACAT-3′ |
|  | Reverse | 5′-CGAGTAGGACATGCTGTAGGT-3′ |
| SOX9 | Forward | 5′-TCCTCAGGCTTTGCGATTT-3′ |
|  | Reverse | 5′-TGCTCGGGCACTTATTGG-3′ |
